# Effectiveness of lifestyle interventions for preventing or managing the adverse cardiometabolic and other physical health effects of antipsychotic medications in children and adolescents: systematic review and meta-analysis

**DOI:** 10.1192/bjo.2025.10919

**Published:** 2025-12-22

**Authors:** Patrick J. Hawker, Jessica Bellamy, Tsz Ying Wong, Catherine McHugh, Philip Ward, Amanda Wood, Bruce Tonge, Katrina Williams, Mark Bellgrove, Tim J. Silk, Vicki Anderson, Farah Akram, Valsamma Eapen

**Affiliations:** Discipline of Psychiatry & Mental Health, School of Clinical Medicine, University of New South Wales, Sydney, New South Wales, Australia; Mindgardens Neuroscience Network, Sydney, New South Wales, Australia; School of Medical, Indigenous and Health Sciences, Faculty of Science, Medicine & Health, University of Wollongong, New South Wales, Australia; Department of Developmental Disability Neuropsychiatry (3DN), School of Clinical Medicine, University of New South Wales, Sydney, New South Wales, Australia; Murdoch Children’s Research Institute (MCRI), Melbourne, Victoria, Australia; Department of Psychology, Faculty of Health/School of Psychology, Deakin University, Melbourne, Victoria, Australia; Department of Psychiatry, Psychiatry Monash Health, Monash University, Melbourne, Victoria, Australia; Department of Paediatrics, Monash University, Melbourne, Victoria, Australia; School of Psychological Sciences, Monash University, Melbourne, Victoria, Australia; Centre for Social and Early Emotional Development and School of Psychology, Deakin University, Burwood, Victoria, Australia; Department of Psychology, Melbourne School of Psychological Sciences, The University of Melbourne, Victoria, Australia

**Keywords:** Neuropsychiatry, psychosocial interventions, psychopharmacology, lifestyle intervention, psychotropic medications

## Abstract

**Background:**

An increasing number of children and adolescents are prescribed second-generation antipsychotic medications, which may lead to cardiometabolic or other physical health impairments. It is unknown whether lifestyle interventions can prevent or manage these adverse effects.

**Aims:**

To evaluate the effectiveness of lifestyle interventions for preventing or managing cardiometabolic risks and other adverse physical health outcomes in this population.

**Method:**

Four bibliographic databases were searched up to February 2024. Randomised controlled trials reporting a physical health outcome of children or adolescents (aged 6–17 years) taking antipsychotics and participating in a lifestyle intervention compared with treatment as usual (TAU) were eligible for inclusion. The Cochrane Risk of Bias 2 tool was used to assess risk of bias. Data were synthesised via a random-effects meta-analysis and narrative synthesis.

**Results:**

Four studies with a total of 370 participants were included. Most (75%) had a high risk of bias. Lifestyle interventions resulted in moderate but statistically non-significant reductions in participants’ body mass index (standard mean difference −0.70, 95% CI: −1.70 to 0.31) compared with TAU. Some studies reported improvements in other physical health outcomes favouring the intervention, although findings were inconsistent and varied across different measures. Reporting of secondary indicators of physical health, including participant or family health behaviours, was limited.

**Conclusions:**

The effectiveness of lifestyle interventions for preventing or managing the cardiometabolic risk and other adverse physical health outcomes in this population is unclear due to the limited number of available trials, small samples and high risk of bias. Larger trials are needed.

Children and adolescents are increasingly prescribed antipsychotic medications. In 2014, the prevalence of antipsychotic use among persons aged 0–19 years ranged from 0.5 to 30.8 per 1000 individuals (0.05–0.38%) globally.^1^ In England, the prevalence of antipsychotic prescriptions for children and adolescents doubled, from 0.057% in 2000 to 0.105% in 2019.^2^ Increases of 137 and 110% were reported in the Netherlands and Denmark, respectively, from 2000 to 2010.^3^ In Canada, the prevalence of antipsychotic use in children and adolescents increased about fourfold over a similar period.^4^ A comprehensive study of data obtained from 16 countries, from 2005 to 2014, reported increased antipsychotic use in children and adolescents in 14 of the 16 countries examined.^1^ It is likely that this global increase is related to a surge in off-label prescribing practices.^5^ This includes the prescription of antipsychotic medications to children and adolescents for anxiety or mood disorders, neurodevelopmental conditions and conduct and behavioural problems.^6–11^

Antipsychotic medications carry a significant risk of adverse physical health effects.^7,12,13^ Adverse cardiometabolic effects, such as weight gain and increased risk of type 2 diabetes, are of particular concern in children and adolescents due to the potential for continued complications into adulthood and associated long-term morbidity.^14–16^ Younger age for the use of antipsychotics promotes vulnerability to the adverse cardiometabolic effects of antipsychotics,^12,17–21^ with children and adolescents gaining disproportionately more weight than adults.^21,22^

Additionally, those with neurodevelopmental conditions who are often prescribed antipsychotics^10,11^ are at risk of specific lifestyle-related challenges including heightened sedentary behaviour,^23^ poor diet and nutrition,^24,25^ disrupted sleep patterns^26^ and frequent tobacco use,^27,28^ all of which could compound the adverse cardiometabolic effects. Indeed, reports suggest that autistic youth are the most susceptible to antipsychotic-induced weight gain,^17^ with as many as one in six potentially receiving treatment with antipsychotic medications.^10^ Weight gain is also likely to impact self-esteem and reduce the willingness of children and adolescents to continue taking antipsychotic medications.^29–32^ Thus, interventions to prevent adverse cardiometabolic effects during childhood have the potential to sustain the use of antipsychotic pharmacotherapy, in addition to preventing continued complications in adulthood.

‘Lifestyle’ interventions are non-pharmacological approaches aimed at modifying behaviour. They are typically multifaceted, including educational, psychotherapeutic or social intervention to modify lifestyle behaviour such as physical activity and dietary habits. Lifestyle interventions are effective at preventing and treating antipsychotic-induced weight gain in adults with serious mental illness.^33–37^ They are also the mainstay for preventing and managing childhood obesity.^38,39^ Given the unique context and characteristics of children and adolescents with neurodevelopmental and other mental health conditions who are regularly prescribed antipsychotics,^10,11,23–28,40^ effective strategies in general paediatrics probably require substantial adaptation. There is limited research on the effectiveness of such strategies, and no systematic analysis of the literature is available. Therefore, this study aims to systematically review and evaluate the effectiveness of lifestyle interventions for preventing or managing cardiometabolic risk and/or other adverse physical health outcomes in children and adolescents taking antipsychotic medications.

Specific research questions were:

- For children and adolescents taking antipsychotic medications, do lifestyle interventions reduce the risk of compromised physical health (any physical health outcome) compared with treatment as usual (TAU)?
- Which individual or combined components of a lifestyle intervention are most effective in reducing the risk of physical health decline?

## Method

This systematic review and meta-analysis was conducted according to the Preferred Reporting Items for Systematic Reviews and Meta-Analyses (PRISMA) guidelines.^41^ The PRISMA checklists are available in Supplementary Material 1.1–1.2 available at https://doi.org/10.1192/bjo.2025.10919. The study protocol was prospectively registered with PROSPERO (no. CRD42019136568) and has been published elsewhere.^42^

### Eligibility criteria

Randomised controlled trials comparing lifestyle interventions with a TAU comparator group were eligible for inclusion if they met the following criteria: the study population included children and adolescents aged 6–17 years (mean age <20 years); ≥55% of the participants were taking an antipsychotic medication (adjusted from the initial criterion of ≥70% in the registered protocol, prior to screening); a physical health outcome (aside from motor development) was measured; and the study was published in English. Lifestyle interventions were defined as any educational, psychotherapeutic, social and behavioural intervention that aims to increase exercise or physical activity, optimise dietary intake, aid nicotine cessation or improve sleep quality and duration.^42^ For comparator groups, TAU was operationalised according to recommended care standards for children and adolescents prescribed antipsychotic medications (i.e. the implementation of metabolic monitoring and/or psychoeducation about weight and lifestyle^43,44^). For a detailed description of the study eligibility criteria, developed using the Population, Intervention, Comparison, Outcomes and Study design (PICOS) framework,^45^ see Supplementary Material 2.

### Information sources and search strategy

Four online bibliographic databases (PubMed, PsycINFO, Embase and Cochrane CENTRAL) were initially searched in March 2023, with searches re-run up to 22 April 2025. The search strategy was developed in PubMed and adapted for the other databases. Search terms were developed using the PICOS framework as a guideline. The search strategy was piloted by identifying a test set of three target papers known to meet the eligibility criteria. Using PubMed unique identifiers, the search strategy was iteratively refined to maximise the sensitivity and specificity for identification of the target articles, until reaching 100% sensitivity. The search strategy conducted in PubMed is available in Supplementary Material 3. The reference lists of relevant studies were searched to identify additional articles.

Following duplicate removal, at least two reviewers (J.B., P.J.H. and/or T.Y.W.). independently conducted title and abstract screening and full-text screening using Covidence systematic review software, version 2014 for Windows (Veritas Health Innovation, Melbourne, Australia; https://www.covidence.org/), with the PICOS framework as an eligibility criterion. Eligibility concerns were resolved by discussion. Interrater reliability for title/abstract screening and full-text screening was moderate (Cohen’s *κ* = 0.41) and excellent (Cohen’s *κ* = 0.87), respectively. Disagreements were primarily related to the study population, due to difficulties in elucidating participants’ antipsychotic medication status during title and abstract screening. Reviewers addressed this by marking records as ‘maybe’ during title and abstract screening when antipsychotic use was suspected (based on participant diagnoses). Medication status was then confirmed during full-text screening.

### Outcomes

While all relevant physical health outcomes were considered, the primary outcome measure was participants’ cardiometabolic health as reflected by the difference in mean body mass index (BMI) change (kg/m^2^) between intervention and TAU groups at the post-intervention time point. BMI was selected as the primary outcome because it is the most robust indicator for identification of individuals whose excess adiposity places them at increased cardiometabolic risk.^46^ It is a validated and replicable proxy measure of adiposity in children and adolescents.^15,47,48^ Additional physical health outcomes included participants’ anthropometric data; blood pressure; any relevant hepatic, metabolic, endocrine and/or haematological data; presence of cardiovascular or respiratory disease; indicators of physical fitness; and physical health-related behaviours.

### Data collection

Data extraction began on 4 January 2024. P.J.H. and J.B. independently extracted study data using a custom template created in Covidence. Physical health outcomes were extracted at all available time points. Study characteristics, including funding information, methods, population characteristics and intervention details, were extracted.

### Data analysis and synthesis

A random-effects meta-analysis model was used to synthesise any physical health outcome reported at a post-intervention time point by three or more studies. The DerSimonian and Laird random-effects model was implemented using the metafor package in R version 4.4.0 for Windows (R Foundation, Vienna, Austria; https://www.r-project.org/).^49^ Standard mean difference (SMD) was calculated from the means and standard deviations comparing intervention and comparator group outcomes at the post-intervention time point, using aggregate data as reported in each study. Authors were contacted on two separate occasions (several weeks apart) when these data were not available. If no response was received, standard deviations were calculated assuming a *z*-score of 1.96 for a 95% confidence interval. The magnitude of effect size was interpreted according to Cohen’s guidelines.^50^ Heterogeneity was assessed using Higgin’s *I* ^2^, with higher values indicating greater heterogeneity.^51^ Substantial heterogeneity was defined as an *I* ^2^ value greater than 75%.^52^ Subgroup and sensitivity analyses were planned *a priori* to explore variations according to intervention characteristics, study risk of bias and participant characteristics. A narrative synthesis was used to summarise evidence of effect where results were not reported in a manner conducive to meta-analysis (insufficient data and/or presented in a format incompatible with other studies).

### Assessments of bias and confidence in evidence

Publication bias was evaluated by visual inspection of funnel plots and Egger and colleagues’ test of asymmetry.^53^ Risk of bias was independently assessed by two reviewers (P.J.H. and J.B.) via the Cochrane risk-of-bias tool for randomised trials version 2 (RoB 2).^54^ Each outcome reported by a minimum of three studies was evaluated. Judgements of ‘low’ or ‘high’ risk of bias, or expression of ‘some concerns’, across domains were generated by the Cochrane algorithm following reviewers’ answers to signalling questions, with discrepancies resolved by discussion. The Grading of Recommendations, Assessment, Development and Evaluations (GRADE)^55^ was used to assess the cumulative body of evidence for the primary outcome measure.

## Results

### Searches

**Fig. 1:**
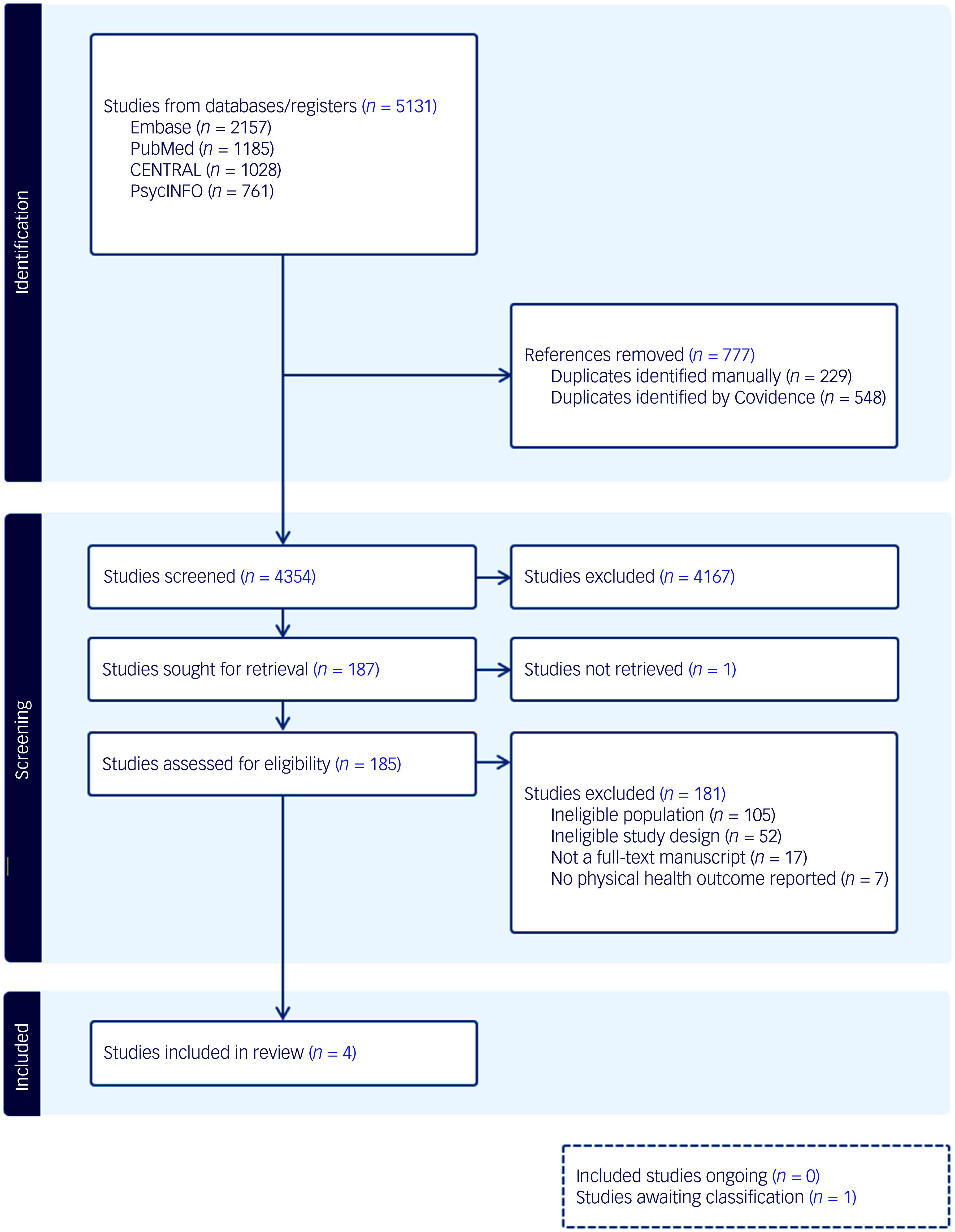
Preferred Reporting Items for Systematic Reviews and Meta-Analyses (PRISMA) flowchart.

Searches revealed 5131 records. Following duplicate removal and title and abstract screening, 185 full texts were assessed for eligibility. Of these, four studies^56–59^ met the inclusion criteria by reporting a physical health outcome measure in populations that included children and adolescents engaged in a lifestyle intervention during antipsychotic pharmacotherapy (see Fig. 1). One study^60^ initially appeared to meet the inclusion criteria but was excluded because it did not contain an eligible comparator group. Another study^61^ was unable to be classified due to the full text being published in a non-English language.

### Participant characteristics

Across all studies, 370 participants were included, with sample sizes ranging from 26 to 203 participants. Of these participants, 204 (55%) received a lifestyle intervention and 166 (45%) received TAU; 221 (60%) participants were male and 149 (40%) were female. The mean age of participants was 14.3 years (range 6–24). Ethnicity data were reported by only one study,^56^ in which 24 participants (12% of their sample) were described as Hispanic or Latino.

In the pooled data from all studies, 340 (91%) participants were taking antipsychotic medications. Data on the type of antipsychotic medication taken were available for 297 (87%) participants. Three studies^56–58^ reported the type of antipsychotic medication taken by participants, with second-generation antipsychotics being prescribed in all cases. Olanzapine, aripiprazole and risperidone were the most frequently reported, representing 209 (70%), 41 (14%) and 21 (7%) instances, respectively. The remaining 26 (9%) antipsychotic prescriptions included paliperidone, lurasidone, quetiapine, amisulpride and ziprasidone.

Across all studies, two^57,58^ reported a proportion of their sample as diagnosed with a psychotic disorder (the proportion of participants with a psychotic disorder across all studies was 41%). Other diagnoses included mood disorders (31%)^56,57^ and neurodevelopmental conditions (i.e. attention-deficit hyperactivity disorder, autism, learning disabilities and intellectual disabilities; 26%).^57,59^ The remaining data included participants with anxiety disorders and other psychiatric diagnoses (2%).^57^

### Study characteristics

One study was a multi-site trial conducted within the USA, Russia, Poland and Germany;^56^ the remaining studies were conducted in the USA,^57^ Australia^58^ and Brazil.^59^ The median duration from baseline to post-intervention was 32 weeks (range 12–52 weeks). One study^58^ included a 6-month follow-up post-intervention. Two studies^56,58^ reported lifestyle interventions to prevent antipsychotic-induced weight gain and/or metabolic complications, while the remaining two studies^57,59^ employed a ‘treatment’ approach to ascertain the physical health benefits of lifestyle interventions delivered to children and adolescents taking medications.

Three studies^56,57,59^ reported the primary outcome measure of participants’ BMI at a post-intervention time point. Laboratory analytes, including hepatic, metabolic, endocrine and haematological data, were reported by all studies. Participants’ waist circumference was reported by three studies.^56,57,59^ Clinically significant weight gain, defined as an increase of ≥7% from baseline weight, was provided by 2 studies.^56,58^ Health behaviours were assessed through standardised questionnaires in two studies.^57,58^ One study^56^ provided data on blood pressure, electrocardiograms and the side-effects of antipsychotic medications. One study^59^ reported on participants’ health-related quality of life as rated by primary caregivers. Further details on study characteristics are provided in Table 1.

**Table 1:** Study characteristics

Study			Population			Intervention			Comparator (TAU)
Name	Time points	*n*	Mean age, years (s.d.)	Female *n* (%)	Antipsychotic medication *n* (%)	Description	Duration	Family-centric	Description
Detke et al^56^	0 and 52 weeks	203 (101 int, 102 cnt)	15.8 (1.5); int: 15.7 (1.5); cnt: 15.9 (1.5)	97 (47.8); int: 46 (45.5); cnt: 48 (47.1)	Olanzapine, 203 (100)	Intense behavioural weight counselling	52 weeks	Yes	Single counselling session
Nicol et al^57^	0 and 16 weeks	26^a^ (19 int, 7 cnt)	13.3 (2.43); int: 13.35 (2.57); cnt: 12.80 (2.21)	21 (44.7); int: 5 (26.32); cnt: 1 (14.29)	Aripiprazole, 12 (46.2) Lurasidone, 1 (3.9) Olanzapine, 2 (7.7) Paliperidone, 1 (3.9) Risperidone, 4 (15.4) Ziprasidone, 4 (15.4) Quetiapine, 4 (15.4)	Family-based social facilitation behavioural weight loss	16 weeks	Yes	Monthly psychoeducation/check-in – no support for self-monitoring and goal-setting
O’Donoghue et al^58^	0 and 12 weeks, 6-month follow-up	77 (38 int, 39 cnt)	19.4 (3.4); int: 19.6 (3.9); cnt: 19.3 (2.9)	38 (49.4); int: 19 (50.0); cnt: 19 (48.7)	Amisulpride, 1 (1.3) Aripiprazole, 29 (37.7) Lurasidone, 4 (5.2) Olanzapine, 4 (5.2) Paliperidone, 7 (9.1) Risperidone, 17 (22.1) Quetiapine, 4 (5.2); no antipsychotic medication, 6 (7.8); missing data, 5 (6.5)	Addition of a physical health nurse to the treatment team	12 weeks	Unclear^b^	Case manager and psychiatrist support
Toscano et al^59^	0 and 48 weeks	90 (46 int, 18 cnt)	int: 8.2 (1.7) cnt: 8.9 (2.0)	8 (12.5)	Unspecified antipsychotic medication, 40 (62.5)	Physical activity programme	48 weeks	Yes	Care as usual within the paediatric centre

TAU, treatment as usual; int, intervention group; cnt, control group.

a. Excludes the ‘non-psychiatric’ group.

b. Parents/guardians/care-givers also consented to the study if the children were aged under 18 years.

#### Intervention and comparator strategies

Three studies^56–58^ employed counselling as the core intervention strategy, and one^59^ used a structured exercise programme. Of the three studies that utilised counselling, all included behavioural modification strategies comprising self-monitoring of physical activity and diet, goal-setting around health behaviours and problem-solving. Three included lifestyle education covering diet/nutrition, physical activity, smoking cessation and the metabolic impacts of antipsychotics. Three^56–58^ included routine physical health monitoring at study visits, and one^58^ included referral to allied health providers (dietitians and physiotherapists) and supported participants’ attendance at smoking cessation and sexual health interventions, when appropriate. The median frequency of participant engagement in lifestyle interventions was once per week (range, weekly to monthly) over a 32-week period (range, 12–52 weeks). Two studies^56,59^ reported the length of intervention sessions with participants, with a median time of 27.5 min per engagement (range, 15–40 min).

Comparator (TAU) groups typically included brief education on weight gain and lifestyle adjustments and/or the implementation of monitoring practices. Further details on intervention and comparator strategies are available in Supplementary Material 4.

### Random-effects meta-analysis

**Fig. 2:**
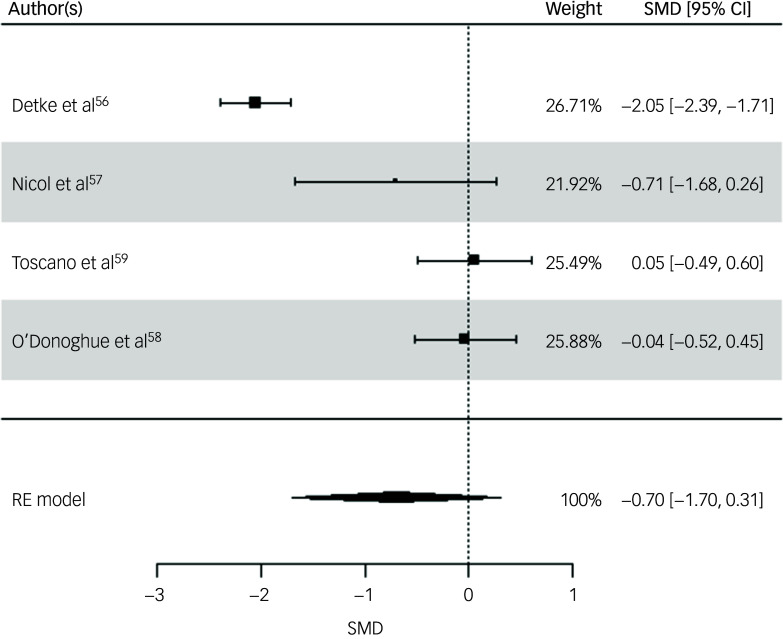
Forest plot for BMI post-intervention. BMI, body mass index; RE, random effects; SMD, standard mean difference.

Three physical health variables, including participants’ BMI, weight and waist circumference, were included in the random-effects model. All four studies were included for participants’ BMI post-intervention. The random-effects model indicated a moderate but statistically non-significant intervention effect on BMI (SMD = −0.70, s.e. = 0.51, *z* = −1.36, *P* = 0.17, 95% CI: −1.70 to 0.31; Fig. 2), with substantial heterogeneity reported (*I* ^2^ = 93%, *t* ^2^ = 0.95, *P* < 0.001). The risk of bias for BMI as rated via RoB 2 was high across all studies.^56,57,59^ Egger’s test indicated no evidence of publication bias (*t* = 1.0852, *P* = 0.39). While interpretation is limited due to the small sample, visual inspection of the funnel plot suggested relative symmetry around the pooled effect size, with study precision inversely related to the magnitude of effect size (Supplementary Material 5.1).

**Fig. 3:**
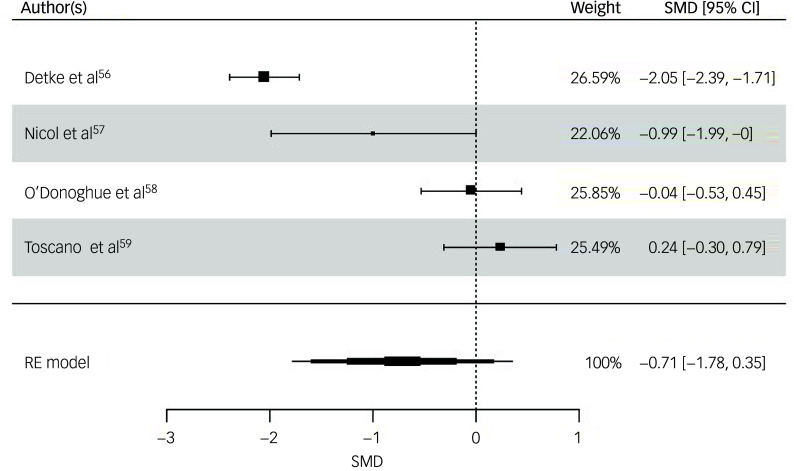
Forest plot for weight post-intervention. RE, random effects; SMD, standard mean difference.

All four studies were included for weight (kg) post-intervention. The random-effects model indicated a moderate but statistically non-significant intervention effect (SMD = −0.71, *z* = −1.32, s.e. = 0.54, *P* = 0.19, 95% CI: −1.78 to 0.35; Fig. 3), with substantial heterogeneity reported (*I* ^2^ = 94%, *t* ^2^ = 1.07, *P* < 0.001). The risk of bias for weight as rated via RoB 2 was ‘high’ for three studies,^56,57,59^ with ‘some concerns’ for O’Donoghue et al.^58^ Egger’s test indicated no evidence of publication bias (*t* = 0.871, *P* = 0.476). While interpretation is limited due to the small sample, visual inspection of the funnel plot of weight suggested a slight pattern of asymmetry, with the most precise study reporting a small effect size and less precise studies indicating a wider variation in effect size (Supplementary Material 5.2).

Three studies were included for waist circumference (centimetres) post-intervention. The random-effects model indicated no effect (SMD = −0, s.e. = 0.1520, *P* = 0.99, 95% CI: −0.30 to 0.30). The risk of bias for waist circumference as rated via RoB 2 was high across all studies.^56,57,59^

The small number of studies limits the ability to perform subgroup analyses that could elucidate factors as sources of heterogeneity for weight and BMI outcomes. The summary statistics for the meta-analysis are presented in Table 2.

**Table 2:** Summary of findings

Outcome category	Number of studies	Sample size			Weighted outcomes			Certainty of the evidence (GRADE)
		Total	Lifestyle intervention	TAU	Lifestyle intervention, mean (s.d.)	TAU, mean (s.d.)	SMD (95% CI)	
BMI (kg/m^2^)	4	353	186	167	1.63 (3.42)	2.33 (2.18)	−0.70 (−1.70, 0.31)	Low
Weight (kg)	4	353	186	167	6.17 (6.44)	8.11 (5.90)	−0.71 (−1.78, 0.35)	Low
Waist circumference (cm)	3	288	153	135	4.93 (3.70)	5.51 (4.51)	−0 (−0.30, 0.30)	Low

TAU, treatment as usual; SMD, standard mean difference; GRADE, Grading of Recommendations, Assessment, Development and Evaluations; BMI, body mass index.

### Narrative synthesis

#### Clinically significant weight gain

Two studies^56,58^ reported the proportion of participants who had experienced clinically significant weight gain (≥7% of baseline weight). Both studies reported no significant differences for clinically significant weight gain between intervention and TAU groups.

#### Presence of metabolic syndrome

One study^58^ included data on the presence of metabolic syndrome, describing no differences in its prevalence between groups either following the 12-week intervention period or at 6-month follow-up.

#### Anthropometric and cardiovascular data

Anthropometric data were collected in three studies,^56,57,59^ with all reporting on participants’ waist circumference and one^56^ assessing blood pressure. No studies reported group-related differences in waist circumference. Detke et al^56^ reported a significant reduction in supine diastolic (*P* = 0.017) and standing systolic pressures (*P* = 0.031) among intervention participants compared with TAU, with no differences observed for supine systolic pressure or standing diastolic pressure.

One study^56^ provided electrocardiogram data, reporting a significant reduction in heart rate (*P* < 0.001) among participants in the intervention group compared with TAU, with no effect observed for QT-interval.

#### Laboratory analytes and imaging

Three studies^56,57,59^ measured an array of hepatic, metabolic, endocrine and/or haematologic analytes. Detke et al^56^ reported no changes for any markers across groups. While Nicol et al^57^ reported a reduction in both hepatic triglyceride content and dual-energy X-ray absorptiometry-measured fat in intervention participants, the effectiveness of the intervention was overestimated by the inclusion of a ‘non-psychiatric’ participant group who engaged in the intervention but were not taking antipsychotic medications. Accounting for this group, calculated differences between intervention and TAU participants were non-significant (*P* > 0.05) for all hepatic, metabolic, endocrine and/or haematologic indicators (Supplementary Material 6). Toscano et al^59^ reported significant reductions in glucose levels and low-density lipoprotein (LDL) cholesterol, as well as an increase in high-density lipoprotein (HDL) cholesterol, among intervention participants compared with TAU (*P* < 0.05; Supplementary Material 6).

#### Adverse effects of antipsychotics

One study^56^ included data on the adverse effects of antipsychotics. No significant between-group differences were reported for any adverse event (*P* > 0.999).

#### Health-related behaviours

Two studies^57,58^ reported on physical health-related behaviours via the use of standardised questionnaires. Physical activity levels were assessed through the International Physical Activity Questionnaire^57^ and the Simple Physical Activity Questionnaire.^58^ Smoking and substance use were evaluated with the Alcohol, Smoking and Substance Involvement Screening Test.^58^ No significant statistical differences were reported between intervention and TAU participants for any domain of health behaviour. No studies reported on health behaviours related to participants’ diet, nutrition or sleep practices. No studies reported on family and/or primary care-giver health behaviours, or on other obesity risk factors linked to the home environment.

#### Health-related quality of life

One study^59^ reported on participants’ health-related quality of life as captured by the parent-completed Child Health Questionnaire.^62^ All of the quality-of-life domains (psychosocial health, physical health, motor profile and autistic traits) were significantly (*P* < 0.05) improved in the intervention group compared with TAU (Supplementary Material 6).

### Quality assessment

**Fig. 4:**
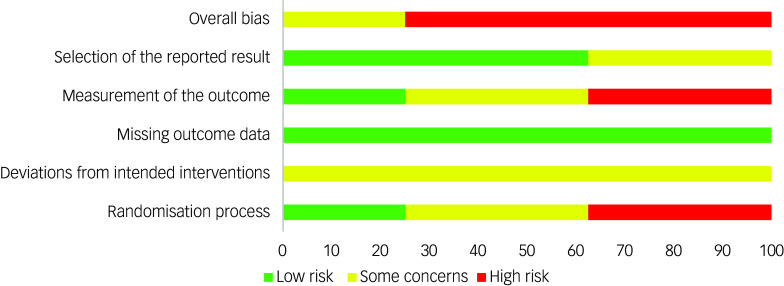
Risk of bias summary.

The summary risk of bias for included studies is available in Fig. 4. The quality of evidence (GRADE) of the primary outcome (BMI) was downgraded to ‘low’ due to substantial heterogeneity and high risk of bias.

## Discussion

To our knowledge, this is the first systematic review and meta-analysis to examine the effectiveness of lifestyle intervention strategies aimed at preventing or managing cardiometabolic risk or other adverse physical health outcomes in children and adolescents taking antipsychotic medications. The random-effects models revealed a moderate but statistically non-significant intervention effect on BMI (kg/m^2^) and mean weight (kg), suggesting that the sample was too small and variable for the observed effect to reach statistical significance. The confidence in aggregate outcomes is limited by the small number of included trials and the presence of substantial heterogeneity. The results should be interpreted with caution due to the low quality of evidence.

Similar meta-analytic reviews in different population groups report large effect sizes of lifestyle interventions on weight that are statistically significant (*P* < 0.05). For example, lifestyle interventions conducted in adults taking antipsychotic medications have been shown to lead to significant reductions in weight compared with TAU (weighted mean difference −0.63 to −2.56 kg/m^2^;^63–65^ SMD −0.2 to −0.24^33^). Obese children without psychiatric diagnoses and participating in a lifestyle intervention may lose anything from 1.30 to 1.49 kg/m^2^ compared with TAU.^66,67^ The current study, however, encompasses a smaller sample size with substantial heterogeneity that probably contributed to the lack of statistical significance observed.

Previous systematic reviews of lifestyle interventions suggest that geographical context, participant populations, intensity and duration of interventions and the variety of diet or exercise regimes employed are potential modulating factors of heterogeneity.^33,35,66^ The present study included research predominantly conducted in Western countries. Participants were diagnostically heterogenous, and the type of antipsychotic medication taken was highly variable (if reported). The type of lifestyle interventions delivered varied in duration, content and delivery, consisting of approaches from behavioural modification and educational strategies to structured physical activity programmes. Thus, heterogeneity probably arises from factors related to participant characteristics and intervention variability.

Our study also aimed to ascertain modulators of treatment effect. The study with negligible effect on participants’ weight and BMI^59^ employed a structured physical exercise programme with no integration of behaviour modification or educational components. That study did not, therefore, follow evidence-based treatment for weight loss including multidisciplinary and behaviour modification,^39,68^ and may not have been intended to stimulate weight change. The study with the largest effect on weight and BMI^56^ employed a prevention approach incorporating behaviour-modification strategies, lifestyle (diet and physical activity) education and physical health monitoring over a 12-month period. Participants in that study^56^ were treated with olanzapine, which has a high propensity to cause weight gain.^69^ The findings are largely consistent with reports that lifestyle interventions may be more effective when either delivered over long durations (>6–12 months),^33,66^ a prevention approach is taken^63^ or patients are treated with an antipsychotic associated with significant weight gain.^70^ However, the scarcity of studies in the present analysis limits the ability to perform subgroup analyses that could elucidate factors related to treatment effects or sources of heterogeneity. Thus, we are unable to address our secondary research question further.

### Other measures of physical health

Waist circumference is increasingly recognised as an indicator of obesity, metabolic syndrome and cardiovascular disease risk.^71–73^ Interestingly, lifestyle interventions showed no effect on participants’ waist circumference, despite the trend for a reduction in BMI. This is unexpected, because waist circumference typically correlates with BMI measured simultaneously,^72^ as observed in lifestyle interventions delivered to overweight or obese children and adolescents.^74^ It is worth nothing that there are many limitations to relying solely on waist circumference to ascertain cardiometabolic risk in children and adolescents: for example, age- and gender-dependent cut-offs are required, or the children’s height may overestimate or underestimate the degree of central adiposity.^72,75^ Thus, typically, the waist-to-height ratio (WtHR), which avoids these limitations, should be collected to identify cardiometabolic risk in children and adolescents.^75–77^ Unfortunately, no studies have reported outcomes related to WtHR.

Measurements of other hepatic, metabolic, endocrine and haematologic markers suggested unclear effects, which contrasts with reports in other populations.^66^ Only one study^59^ reported significant benefits, showing reductions in glucose levels and LDL cholesterol and an increase in HDL cholesterol among intervention participants compared with TAU. The absence of observed effects in other studies may be attributed to limited sample size, because comparisons against non-psychiatric populations reveal the positive benefits of lifestyle interventions on central adiposity markers.^57^ These findings highlight the need for further research to better understand the variability in responses to lifestyle interventions among different age groups and populations.

While no data were collected on participants’ dietary behaviour, nutrition or sleep practices, several studies have reported on physical activity outcomes. No significant differences between groups were reported, even though behavioural modification is a core strategy of lifestyle interventions. However, participants’ physical activity was captured via questionnaires not validated in child or adolescent populations,^78,79^ suggesting that the results should be interpreted with caution.

For participants supported by a parent or guardian during the study period, factors related to the family and home environment may have influenced their outcomes. Children with obese parents are at a greater risk of obesity compared with those having non-obese parents.^80^ The risk of obesity is thought to be transmitted to the next generation through a complex interaction of genetic, metabolic, behavioural, cultural and environmental factors.^81^ Importantly, modifiable factors such as parental health behaviour and the home environment are predictors of children’s physical activity, sedentary behaviour and dietary intake.^82,83^ In the present study, no trials captured outcomes related to parental behaviour change or the home environment. Given the influence of these factors on children’s health behaviour, future studies should aim to assess obesogenic factors related to the home environment and parental health behaviours alongside those of children and adolescents participating in lifestyle interventions.

The report from one study^59^ suggesting that lifestyle interventions may positively impact health-related quality of life is promising, but additional data are needed to confirm any treatment effect. Some studies have shown a significant relationship between quality of life and health-related behaviours.^84,85^ Therefore, routine data collection should include assessments of quality of life for both children and their families undertaking lifestyle interventions. Such data may provide a better understanding of predictors of success and potential incidental benefits.

### Clinical implications

While no studies have reported a weight loss of ≥7% in participants’ weight, the potential for even modest weight reductions in the developmental period to confer significant health advantages should be appreciated. However, the trends for weight loss were not statistically significant. Previous reports suggest that lifestyle interventions alone are insufficient to prevent BMI increase in children and adolescents with serious mental illness (schizophrenia spectrum disorder, bipolar spectrum disorder or psychotic depression^60^). In this cohort, adding metformin or switching to a lower-risk antipsychotic reduced the risk of overweigh/obesity compared with lifestyle intervention alone.^60^ Thus, the clinical significance of lifestyle interventions for children and adolescents prescribed an antipsychotic is unclear and requires further investigation.

### Limitations

This review is limited by several factors. The search process revealed moderate interrater reliability during title and abstract screening, with the diagnostic heterogeneity of children and adolescents taking antipsychotic medications posing barriers to making accurate judgements about participants’ medication status without a full-text report. Additionally, the small sample size and high heterogeneity of the meta-analysis limit the confidence in aggregate outcomes. With only four studies, the DerSimonian and Laird random-effects model provides imprecise estimates of between-study variance (*τ* ^2^), and measures of heterogeneity (*I* ^2^) are unstable. As a result, the pooled effect is sensitive to single-study influence and is best interpreted as a cautious summary of the observed evidence rather than a definitive estimate. The strength of the body of evidence was rated as low, suggesting that the true effect might be markedly different from that estimated.^55^ Finally, the planned subgroup analysis could not be undertaken due to the limited number of trials available. Future studies with more trials should aim to perform such analyses. It is possible that a less rigorous eligibility criterion (i.e. inclusion of observational studies) may have provided additional insights.

### Implication for future research

The scarcity of available literature juxtaposes the rapidly increasing prescription of antipsychotic medications to children and adolescents. This gap highlights an urgent need for future research to implement and evaluate lifestyle interventions for the prevention or management of antipsychotic-induced weight gain and associated cardiometabolic risks. Effort should be made to establish programmes, in partnership with primary care or other providers in rural/remote or socioeconomically disadvantaged regions where off-label prescribing practices are prevalent and there is restricted access to non-pharmacological interventions.^5,86,87^

Additionally, studies should ensure the use of age-appropriate, validated measurement of cardiometabolic risk, health behaviours (i.e. physical activity, dietary habits and sleep patterns) and quality of life of children and adolescents and their parents or guardians, in addition to obesogenic factors related to the home environment. The inclusion of extended follow-ups will help determine the sustainability of these interventions, recognising that adopting healthy behaviours during the developmental period can have substantial long-term benefits when maintained.

## Supporting information

Hawker et al. supplementary material 1 Hawker et al. supplementary material

Hawker et al. supplementary material 2 Hawker et al. supplementary material

Hawker et al. supplementary material 3 Hawker et al. supplementary material

## Data Availability

The data that support the findings of this study, and the analytic code associated with the manuscript, are available from the corresponding author, V.E., upon reasonable request. The research materials associated with the manuscript are available in the supplementary material.

## Supplementary material

The supplementary material is available online at http://doi.org/10.1192/bjo.2025.10919
