# Supplementary material for: Effectiveness of lifestyle interventions for preventing or managing the adverse cardiometabolic and other physical health effects of antipsychotic medications in children and adolescents: systematic review and meta-analysis
Source: BJPsych Open. 2025 Dec 22;12(1):e21. doi: 10.1192/bjo.2025.10919 (PMC12724091; doi:10.1192/bjo.2025.10919)
Supplement: Hawker et al. supplementary material 3 — Hawker et al. supplementary material [file S2056472425109198sup003.docx]

**Supplementary Material S2:** PICOS Table

| PICOS Item | **Eligibility Criteria** |
| --- | --- |
| Population | Youth aged 6 to 17 years taking antipsychotic medications.^†*^ |
| Interventions | All interventions that incorporated a ‘lifestyle’ intervention component and aimed to improve physical health outcomes were eligible. This included any educational, psychotherapeutic, social and behavioural intervention that aimed to increase exercise or physical activity, optimise dietary intake, aid nicotine cessation or improve sleep quality and duration. |
| Comparison | All relevant control interventions were eligible (i.e., treatment as usual/usual care, placebo, no treatment, waiting list). |
| Outcomes | Physical health outcomes that were eligible: (1) Anthropometric measures, including weight, height, waist circumference, or BMI percentile; (2) Blood pressure; (3) Metabolic or biological markers, including glucose and lipid levels, HbA1c, C-reactive protein or other relevant blood and serum markers; (4) Presence of cardiovascular or respiratory disease; (5) Physical health behaviour, including physical activity levels, smoking/vaping behaviour, dietary intake, sleep quality and duration, engagement in treatment and attendance; (6) Indicators of physical fitness, including aerobic capacity (i.e., VO_2_ Max), and muscle strength; (7) Physical health-related quality of life; and (8) Side effects of antipsychotics, including adverse drug reactions.  Physical health outcomes that were not eligible: motor development. |
| Setting | All settings were eligible: primary and secondary care, hospital (inpatient or outpatient), community and school-based service provisions or remote (digital application-based or telehealth/web health services). |
| Study Design & Characteristics | Applicable randomised controlled trials published in the English language were eligible. All years were considered, and no date restrictions were applied. Pseudorandomised control trials, comparative studies with concurrent controls, case series and cohort studies were not eligible. Conference abstracts, dissertations/theses, papers that are not peer-reviewed and papers published in a language other than English were not eligible. |

***Note:*** *BMI, Body mass index; HbA1c, Haemoglobin A1c; PICOS, Population, Intervention, Comparison, Outcomes and Study design; RCT, Randomised controlled trial.*

*^†^ Studies were excluded if their sample’s mean age was < 20 years.*

*^*^ For a study to be eligible, at least 55% of participants needed to be prescribed an antipsychotic medication. This was adjusted from the initial criteria of ≥ 70% of participants in the registered protocol, prior to screening.*

**Supplementary Material S3:** Search Terms for Bibliographic Databases.

| Concept | PubMed | Cochrane Central | Embase (Ovid) | PsycINFO (Ovid) |
| --- | --- | --- | --- | --- |
| *Children and adolescents #1* | "Child"[MeSH Terms] OR "Adolescent"[MeSH Terms] OR "youth"[Text Word] OR "infant"[Text Word] OR "juvenile"[Text Word] OR "minor"[Text Word] OR "young person"[Text Word] OR "youngster"[Text Word] OR "kid"[Text Word] OR "kids"[Text Word] OR "child*"[Text Word] OR "adolesc*"[Text Word] OR "teen*"[Text Word] | "youth" or "infant" or "juvenile" or "minor" or "young person" or "youngster" or "kid" or "kids" or "child*" or "adolesc* or teen*" | "youth" or "infant" or "juvenile" or "minor" or "young person" or "youngster" or "kid" or "kids" or "child*" or "adolesc* or teen*" | "youth" or "infant" or "juvenile" or "minor" or "young person" or "youngster" or "kid" or "kids" or "child*" or "adolesc* or teen*" |
| *Population (diagnosis of mental health condition likely to be prescribed antipsychotic medications). #2* | "Neurodevelopmental Disorders"[MeSH Terms:noexp] OR "Attention Deficit Disorder with Hyperactivity"[MeSH Terms] OR "Developmental Disabilities"[MeSH Terms] OR "Intellectual Disability"[MeSH Terms:noexp] OR “intellectual disabilit*”[Text Word] OR “intellectual impairment*”[Text Word] OR “intellectual delay*”[Text Word] OR “learning disabilit*”[Text Word] OR “learning impairment*”[Text Word] OR “learning delay*”[Text Word] OR “developmental disabilit*”[Text Word] OR “developmental delay”[Text Word] OR "Child Development Disorders, Pervasive"[Mesh] OR "schizophrenia, childhood"[MeSH Terms] OR "Tic Disorders"[MeSH Terms] OR "autism*"[Text Word] OR "autistic*"[Text Word] OR "Kanner's Syndrome"[Text Word] OR "neurodevelopmental*"[Text Word] OR "ADHD"[Text Word] OR "attention defici*"[Text Word] OR "pervasive developmental disorder"[Text Word] OR "developmental disabilit*"[Text Word] OR "intellectual disabilit*"[Text Word] OR "schizophrenia"[Text Word] OR "psychosis"[Text Word] OR "tourette*"[Text Word] OR "tic disorder"[Text Word] OR “asperger*”[Text Word] OR "Bipolar and Related Disorders"[Mesh] OR “bipolar*”[Text Word] | "intellectual disabilit*" or "intellectual impairment*" or "intellectual delay*" or "learning disabilit*" or "learning impairment*" or "learning delay*" or "developmental disabilit*" or "developmental delay" or "autism*" or "autistic*" or "Kanners Syndrome" or "neurodevelopmental*" or "ADHD" or "attention defici*" or "pervasive developmental disorder" or "developmental disabilit*" or "intellectual disabilit*" or "schizophrenia" or "psychosis" or "tourette*" or "tic disorder" or "asperger*" or "bipolar*" | "intellectual disabilit*" or "intellectual impairment*" or "intellectual delay*" or "learning disabilit*" or "learning impairment*" or "learning delay*" or "developmental disabilit*" or "developmental delay" or "autism*" or "autistic*" or "Kanners Syndrome" or "neurodevelopmental*" or "ADHD" or "attention defici*" or "pervasive developmental disorder" or "developmental disabilit*" or "intellectual disabilit*" or "schizophrenia" or "psychosis" or "tourette*" or "tic disorder" or "asperger*" or "bipolar*" | "intellectual disabilit*" or "intellectual impairment*" or "intellectual delay*" or "learning disabilit*" or "learning impairment*" or "learning delay*" or "developmental disabilit*" or "developmental delay" or "autism*" or "autistic*" or "Kanners Syndrome" or "neurodevelopmental*" or "ADHD" or "attention defici*" or "pervasive developmental disorder" or "developmental disabilit*" or "intellectual disabilit*" or "schizophrenia" or "psychosis" or "tourette*" or "tic disorder" or "asperger*" or "bipolar*" |
| *Population (people taking antipsychotic medications). #3* | "antipsychotic*"[Text Word] OR "Antipsychotic Agents"[MeSH Terms] OR "second generation antipsychotic*"[Text Word] | "antipsychotic*" or "second generation antipsychotic*" | "antipsychotic*" or "second generation antipsychotic*" | "antipsychotic*" or "second generation antipsychotic*" |
| *#2 OR #3* |  |  |  |  |
| *#1 AND #4* |  |  |  |  |
| *Lifestyle intervention #6* | “lifestyle intervention”[Text Word] OR “healthy living”[Text Word] OR “healthy lifestyle”[Text Word] OR “lifestyle education”[Text Word] OR “behavioral intervention”[Text Word] OR “behavioural intervention”[Text Word] OR "Behavior Therapy"[Mesh:NoExp]OR "behavioural change"[Text Word] OR "behavioral change"[Text Word] OR “behaviour change”[Text Word] OR “behavior change”[Text Word] OR “behaviour modification”[Text Word] OR “behavior modification”[Text Word] OR “behavioural modification”[Text Word] OR“behavioral modification”[Text Word] OR “physical activity”[Text Word] OR “dietary modification”[Text Word] OR “nutrition therapy”[Text Word] OR “physical therapy”[Text Word] OR “nutrition intervention”[Text Word] OR "Diet Therapy"[Mesh:NoExp] OR “weight loss”[Text Word] | "lifestyle intervention" or "healthy living" or "healthy lifestyle" or "lifestyle education" or "behavioral intervention" or "behavioural intervention" or "behavioural change" or "behavioral change" or "behaviour change" or "behavior change" or "behaviour modification" or "behavior modification" or "behavioural modification" or "behavioral modification" or "physical activity" or "dietary modification" or "nutrition therapy" or "physical therapy" or "nutrition intervention" or "weight loss" | "lifestyle intervention" or "healthy living" or "healthy lifestyle" or "lifestyle education" or "behavioral intervention" or "behavioural intervention" or "behavioural change" or "behavioral change" or "behaviour change" or "behavior change" or "behaviour modification" or "behavior modification" or "behavioural modification" or "behavioral modification" or "physical activity" or "dietary modification" or "nutrition therapy" or "physical therapy" or "nutrition intervention" or "weight loss" | "lifestyle intervention" or "healthy living" or "healthy lifestyle" or "lifestyle education" or "behavioral intervention" or "behavioural intervention" or "behavioural change" or "behavioral change" or "behaviour change" or "behavior change" or "behaviour modification" or "behavior modification" or "behavioural modification" or "behavioral modification" or "physical activity" or "dietary modification" or "nutrition therapy" or "physical therapy" or "nutrition intervention" or "weight loss" |
| *Randomised controlled trial (Cochrane sensitivity and specificity maximising filter) #7* | ("randomized controlled trial"[Publication Type] OR "controlled clinical trial"[Publication Type] OR "randomized"[Title/Abstract] OR "placebo"[Title/Abstract] OR "clinical trials as topic"[MeSH Terms:noexp] OR "randomly"[Title/Abstract] OR "trial"[Title]) NOT ("animals"[MeSH Terms] NOT "humans"[MeSH Terms]) |  | (Randomized controlled trial/ or Controlled clinical study/ or random$.ti,ab. or randomization/ or intermethod comparison/ or placebo.ti,ab. or (compare or compared or comparison).ti. or ((evaluated or evaluate or evaluating or assessed or assess) and (compare or compared or comparing or comparison)).ab. or (open adj label).ti,ab. or ((double or single or doubly or singly) adj (blind or blinded or blindly)).ti,ab. or double blind procedure/ or parallel group$1.ti,ab. or (crossover or cross over).ti,ab. or ((assign$ or match or matched or allocation) adj5 (alternate or group$1 or intervention$1 or patient$1 or subject$1 or participant$1)).ti,ab. or (assigned or allocated).ti,ab. or (controlled adj7 (study or design or trial)).ti,ab. or (volunteer or volunteers).ti,ab. or human experiment/ or trial.ti.) not (((random$ adj sampl$ adj7 ("cross section$" or questionnaire$1 or survey$ or database$1)).ti,ab. not (comparative study/ or controlled study/ or randomi?ed controlled.ti,ab. or randomly assigned.ti,ab.)) or (Cross-sectional study/ not (randomized controlled trial/ or controlled clinical study/ or controlled study/ or randomi?ed controlled.ti,ab. or control group$1.ti,ab.)) or (((case adj control$) and random$) not randomi?ed controlled).ti,ab. or (Systematic review not (trial or study)).ti. or (nonrandom$ not random$).ti,ab. or "Random field$".ti,ab. or (random cluster adj3 sampl$).ti,ab. or ((review.ab. and review.pt.) not trial.ti.) or ("we searched".ab. and (review.ti. or review.pt.)) or "update review".ab. or (databases adj4 searched).ab. or ((rat or rats or mouse or mice or swine or porcine or murine or sheep or lambs or pigs or piglets or rabbit or rabbits or cat or cats or dog or dogs or cattle or bovine or monkey or monkeys or trout or marmoset$1).ti. and animal experiment/) or (Animal experiment/ not (human experiment/ or human/))) | (double-blind or random: assigned or control).tw. |
| #5 and #6 and #7 |  |  |  |  |

**Supplementary Table S4:** Intervention and Comparator Strategies and Components.

| Study | Intervention | | | | Comparator (TAU) |
| --- | --- | --- | --- | --- | --- |
|  | *Strategy* | *Aim* | *Components* | *Engagements* | *Components* |
| Detke *et al*., 2016 | Counselling | Prevention of weight gain | Delivered by trained clinical site personnel:   - Education on healthy eating/diet and physical activity adapted from Smart Moves^TM^ weight management program. - Behaviour modification strategies (self-monitoring of food/beverage intake and physical activity by a food log and pedometer, respectively; goal setting and problem-solving barriers to success) - Physical health monitoring. | At least 15 minutes – twice in week 1; weekly through week 4; every 2 weeks through week 8; and then every 4 weeks through week 52 | - Single counselling session at time of randomisation - Basic information on healthy eating and exercise - Physical health monitoring |
| Nicol *et al*., 2019 | Counselling | Improvement of adiposity and cardiometabolic risk | Delivered by interventionist:   - Education on energy balance/nutrition and physical activity using the Stoplight Traffic Light Plan - Behaviour modification strategies such as self-monitoring of activity, food intake, and sleep, goal setting around health behaviours and problem solving - Physical health monitoring | Weekly | - Access to educational materials and check-in with interventionalist - Physical health monitoring - No support for self-monitoring and goal setting (self-directed) |
| O’Donoghue *et al*., 2022 | Counselling | Prevention of weight gain | Delivered by physical health nurse:   - Education about diet, physical activity, and metabolic impacts of antipsychotics - Behaviour modification strategies including problem solving barriers to physical activity - Physical health monitoring - Dietician and physiotherapist referral - Support for attending smoking cessation and sexual health interventions (where applicable) | Weekly – length not described | - Access to interventions and services supported by case manager and/or psychiatrist - Physical health monitoring - No contact with physical health nurse |
| Toscano *et al*., 2018 | Exercise Program | Improvement of metabolic profile | - Upper and lower limb strength and coordination | 40 minutes twice a week | - Care as usual within the paediatric centre |

**Supplementary Figure S5.1:** Funnel Plot for BMI.


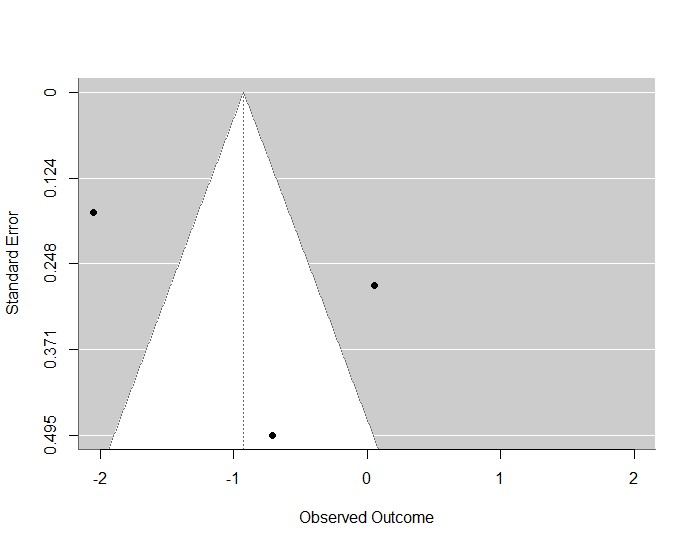


**Supplementary Figure S5.2:** Funnel Plot for Weight.

**
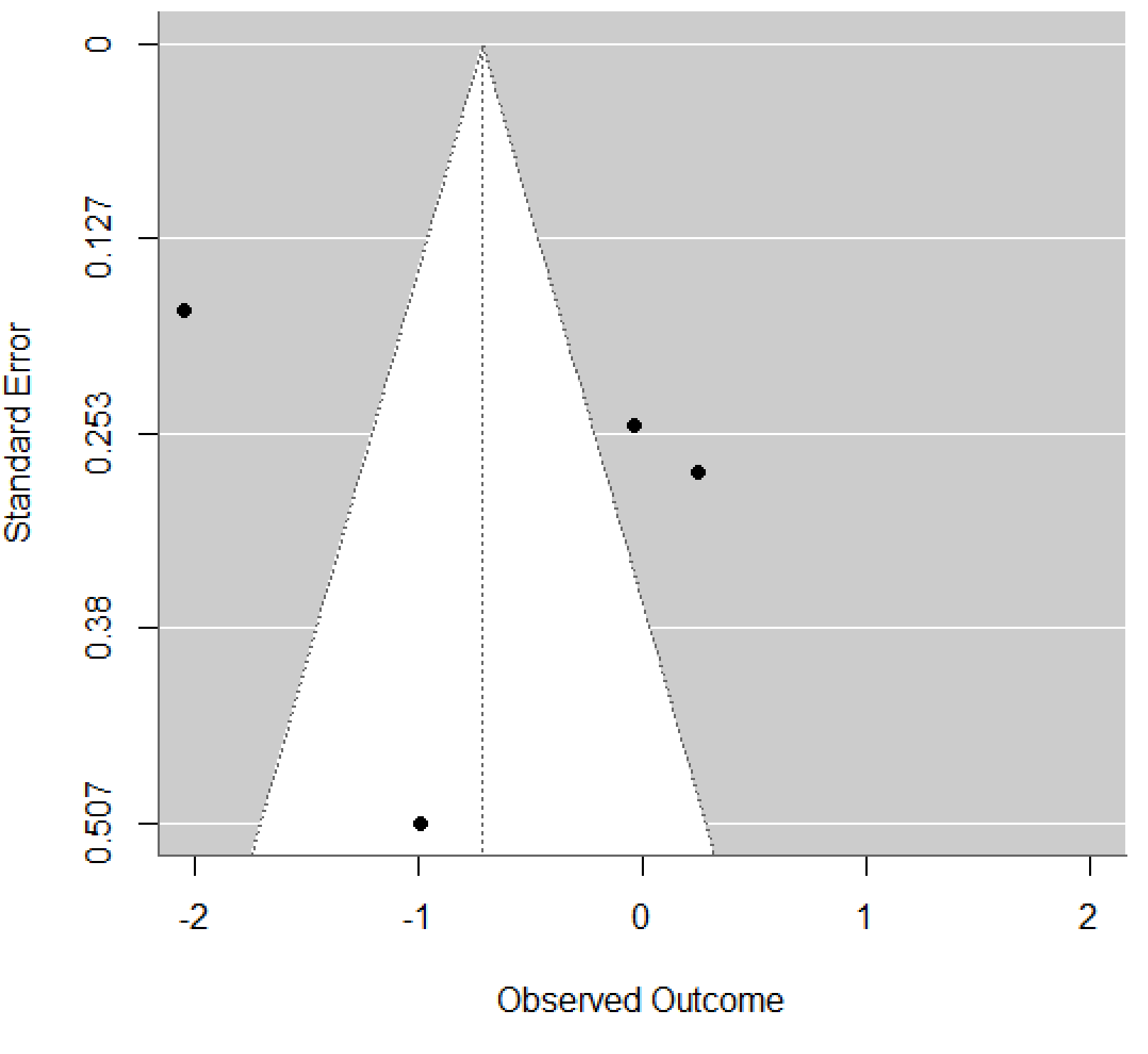
**

**Supplementary Table S6:** Additional Physical Health Outcomes Reported by Studies.

| Study | Outcome^a^ | | |  | Intervention | Control | *p*-Value |
| --- | --- | --- | --- | --- | --- | --- | --- |
| Detke *et al*., 2016 | *Clinically significant (≥ 7%) weight gain* | | | *%* | 63.4 | 56.9 | NS |
| O’Donoghue *et al*., 2022 | *Clinically significant (≥ 7%) weight gain* | | | *n (%)* | 9 (27.3) | 11 (34.4) | 0.54 |
|  | *6-month follow-up* | | |  | 11 (40.7) | 15 (44.1) | 0.79 |
|  | *Metabolic syndrome – present* | | |  | 3 (17.6) | 2 (11.1) | 0.58 |
|  | *Metabolic syndrome – not present* | | |  | 14 (82.4) | 16 (88.9) |  |
|  | *6-month follow-up (metabolic syndrome – present)* | | |  | 2 (20.0) | 3 (25.0) | 0.78 |
|  | *6-month follow-up (metabolic syndrome – not present)* | | |  | 8 (80.0) | 9 (75.0) |  |
| Nicol *et al*., 2019 | DEXA-measured fat (kg) | | | *mean change (SD)* | -0.43 (3.13) | 0.76 (2.05) | 0.377^b^ |
|  | CIMT (cm) | | |  | 0.0032 (0.0098) | -0.0014 (0.0045) | 0.271^b^ |
|  | *PDFF-measured hepatic triglyceride content (%)* | | |  | -0.41 (4.95) | -0.62 (1.57) | 0.916^c^ |
| Detke *et al*., 2016 | *Blood pressure (mmHg)* | | *Supine systolic* | *LS mean change (SE)* | 0.5 (1.1) | 2.5 (1.1) | 0.185 |
|  |  |  | *Supine diastolic* |  | -0.4 (0.8) | 2.4 (0.8) | * 0.017 |
|  |  |  | *Standing systolic* |  | 0.6 (1.0) | 3.7 (1.0) | * 0.031 |
|  |  |  | *Standing diastolic* |  | -0.2 (0.9) | 1.0 (0.9) | 0.318 |
|  | *Heart rate (bpm)* | | |  | 1.3 (1.3) | 7.7 (1.3) | * <0.001 |
|  | *Fridericia-corrected QT interval (milliseconds)* | | |  | 2.0 (1.6) | 0.9 (1.6) | 0.632 |
|  | *Alanine aminotransferase (ALT; U/L)* | | |  | 2.0 (1.8) | 5.6 (1.8) | 0.158 |
|  | *Aspartate aminotransferase (AST; U/L)* | | |  | 0.2 (1.0) | 2.3 (1.0) | 0.121 |
|  | *Gamma glutamyl transferase (GGT; U/L)* | | |  | 0.1 (1.6) | 3.4 (1.6) | 0.136 |
|  | *Total bilirubin (µmol/L)* | | |  | -1.4 (0.3) | -1.1 (0.3) | 0.410 |
| Detke *et al*., 2016 | *Fasting glucose* | *mmol/L* | | *LS mean change (SE)* | 0.1 (1.0) | 0.1 (0.1) | 0.724 |
| Nicol *et al*., 2019 |  | *mg/dL* | | *mean change (SD)* | -1.07 (6.86) | -0.83 (8.89) | NS |
| Toscano *et al*., 2018 |  |  |  | *mean change [CI]* | -1.5 [-4.7 to 1.6] | 2.8 [0.2 to 5.5] | * 0.045^c^ |
| Detke *et al*., 2016 | *Total cholesterol* | *mmol/L* | | *LS mean change (SE)* | -0.02 (0.1) | 0.04 (0.1) | 0.486 |
| Nicol *et al*., 2019 |  | *mg/dL* | | *mean change (SD)* | -1.93 (22.01) | -17.83 (18.15) | NS |
| Toscano *et al*., 2018 |  |  |  | *mean change [CI]* | -10.1 [-19.0 to 1.3] | 0.2 [-7.2 to 7.7] | 0.238^c^ |
| Detke *et al*., 2016 | *HDL cholesterol* | *mmol/L* | | *LS mean change (SE)* | -0.1 (0.02) | -0.1 (0.02) | 0.206 |
| Nicol *et al*., 2019 |  | *mg/dL* | | *mean change (SD)* | 0.47 (6.48) | -5.50 (5.68) | NS |
| Toscano *et al*., 2018 |  |  |  | *mean change [CI]* | 5.2 [2.2 to 8.1] | 0.9 [-1.6 to 3.4] | * 0.034^c^ |
| Detke *et al*., 2016 | *LDL cholesterol* | *mmol/L* | | *LS mean change (SE)* | 0.0 (0.1) | 0.1 (0.1) | 0.390 |
| Nicol *et al*., 2019 |  | *mg/dL* | | *mean change (SD)* | -6.00 (19.16) | -19.00 (14.11) | NS |
| Toscano *et al*., 2018 |  |  |  | *mean change [CI]* | -7.7 [-14.5 to -0.9] | 2.0 [-3.8 to 7.8] | * 0.038^c^ |
| Detke *et al*., 2016 | *Triglycerides* | *mmol/L* | | *LS mean change (SE)* | 0.4 (0.1) | 0.3 (0.1) | 0.279 |
| Nicol *et al*., 2019 |  | *mg/dL* | | *mean change (SD)* | 15.20 (67.91) | 33.00 (30.11) | NS |
| Toscano *et al*., 2018 |  |  |  | *mean change [CI]* | 33.1 [-8.6 to 74.9] | -38.1 [-73.5 to -2.72] | 0.052^c^ |
| Detke *et al*., 2016 | *Insulin (µIU/mL)* | | | *LS mean change (SE)* | 7.3 (2.4) | 5.0 (2.4) | 0.489 |
|  | *Prolactin (µg/L)* | | |  | -0.6 (1.1) | -1.7 (1.1) | 0.501 |
|  | Leukocytes (bill/L) | | |  | -0.3 (0.2) | -0.1 (0.2) | 0.560 |
|  | Neutrophils (bill/L) | | |  | -0.4 (0.2) | -0.2 (0.2) | 0.322 |
| Nicol *et al*., 2019 | *Physical activity (IPAQ; MET-minutes/week)* | | | *mean change (SD)* | 1053.92 (2544.03) | 597.83 (822.59) | NS |
| O’Donoghue *et al*., 2022 | *Physical activity levels (SIMPAQ)^b^* | | | *median (IQR)* | 2.4 (1.4–4.0) | 1.5 (0.4–3.5) | 0.13 |
|  | *6-month follow-up* | | |  | 2.20 (1.5–3.8) | 2.0 (0.6–5.0) | 0.57 |
|  | *Tobacco smoking (daily smokers)* | | | *n (%)* | 11 (35.5) | 10 (32.3) | 0.78 |
|  | *Tobacco smoking (non-smokers)* | | |  | 20 (64.5) | 31 (67.31) |  |
|  | *6-month follow-up (daily smokers)* | | |  | 9 (30.0) | 11 (40.7) | 0.40 |
|  | *6-month follow-up (non-smokers)* | | |  | 21 (70.0) | 16 (59.3) |  |
| Toscano *et al*., 2018 | *Autistic traits* | | *CHQ-PF50* | *mean change [CI]* | -8.1 [-12.2 to -4] | -1.4 [-4.9 to 2.1] | * 0.018 |
|  | *Motor profile* | |  |  | -2.4 [-3.3 to -1.5] | 0.1 [-0.7 to 0.9] | * < 0.001 |
|  | *Physical health* | |  |  | 13.3 [7.7 to 18.9] | 3.1 [-1.8 to 8.1] | * 0.009 |
|  | *Psychosocial health* | |  |  | 15.2 [9.8 to 20.7] | -2.4 [-7.1 to 2.4] | * < 0.001 |

*^a^ Outcomes are reported at the post-intervention timepoint unless stated otherwise.*

*^b^ Level of physical activity was calculated by adding the average daily hours of walking (question 3), doing sport or exercise (question 4) and other physical activities (question 5).*

*^c^ P-values were calculated using Welch's t-test to compare the means of two independent groups with unequal variances and sample sizes. This method was chosen to account for the removal of the non-psychiatric control group, ensuring an accurate comparison between the eligible intervention and comparator groups.*

*^d^ P-values were calculated using Welch's t-test to compare the means of two independent groups with unequal variances and sample sizes. Standard deviations were estimated from the 95% confidence intervals provided for each group.* *This method was chosen because only reported effect sizes were available without a description of the test statistic.*

***Note:*** **, p < 0.05; -, data not available; CHQ-PF50, Portuguese version of the Child Health Questionnaire; CI, Confidence Interval; CIMT, carotid intima media thickness; IPAQ, International Physical Activity Questionnaire; LS, least-scores; NS, not significant; PDFF, proton density fat fraction; SE, standard error.*
